# Save the planet with green industries using algae

**DOI:** 10.1371/journal.pbio.3002061

**Published:** 2023-03-27

**Authors:** Peter J. Ralph, Mathieu Pernice

**Affiliations:** Climate Change Cluster, Faculty of Science, University of Technology Sydney, Ultimo, NSW Australia

## Abstract

We can use photosynthesis to capture carbon and make industries greener. Algae-driven carbon capture and manufacturing offer the potential for reducing CO_2_ emissions while also producing commodities such as bioplastics.

Society has recognised the urgent need to transition our entire way of existence from complete dependence on fossil-derived carbon to replacing it with atmospheric carbon. Over the past 200 years, our industrial development has been driven by cheap and accessible sources of energy, and in doing so, we have liberated vast quantities of fossil-carbon into the atmosphere. Now that we have access to increasing amounts of renewable energy, we can transition away from fossil carbon as a source of energy. But carbon also has another important role in our society, as a raw material for much of our manufacturing industries. Therefore, to complete our transition away from fossil-derived carbon, we critically need to stop using fossil-derived carbon as a raw material for manufacturing. This is where photosynthetic organisms become important; especially microscopic algae, which can capture atmospheric carbon up to 50 x more efficiently than higher plants [1] and convert it into biomass via photosynthesis. This is one vital tool to address climate change.

Knowing the origin of carbon (i.e., atmospheric vs fossil) is critical to defining its future use, as carbon *per se* is a critical element in society from food to plastics. We need to end our use of fossil-derived carbon and promote the use of atmospheric carbon–who knows, this might even make carbon have a value rather than being seen as a problem for emitters to be taxed. Many governments are currently focused on carbon capture and storage (CCS), a process of capturing and storing carbon, generally emitted from fossil-derived extraction, just before it enters into the atmosphere to help mitigate climate change. Using carbon offsets linked to CCS such as forest biomass and Blue Carbon habitats is important, but eventually, those areas will compete with land needed for food production [2]. As the next step, we need to encourage industry to make products with this carbon captured by photosynthesis and then use those products as vehicles to store carbon. For example, algae-derived plastics can generate an income stream valued at $1,540–8,800 USD/tonne [3]; and this is in addition to the value of the captured carbon. Because of the added value of the products manufactured with this carbon, this approach, called carbon capture and manufacture (CCM), has even more economic potential than CCS.

Recognising the nexus between industry, society and algae is critical if we are to use photosynthesis to capture carbon and make industries greener. With algae-driven CCM, industry can capture atmospheric (or emitted) CO_2_, let the algae cells do the chemical conversion and use this carbon in products needed across our society. For example, we have been working with Young Henrys Brewery to capture their emitted CO_2_ from fermentation, grow algal biomass and return this into commercial bioproducts [4]. This is carbon circularity, the longer our algae-derived carbon consumer product remains intact, the longer the carbon is out of the atmosphere and therefore the more we can mitigate climate change.

A prevalent example of using photosynthesis to make industries greener is the relatively recent plant-based meat industry, which provides many environmental benefits relative to the conventional meat industry, including a significant reduction in CO_2_ emissions. While most of the plant-based meat industry currently relies on crops, predominantly soy, to do the heavy lifting of chemical conversion in bioproducts, algae-driven CCM has great potential and could help further to reduce CO_2_ emissions and land use in the future [5]. However, when used in such food products, the algae-derived carbon product would rapidly be reemitted into the atmosphere. One way to take algae-driven CCM to the next step would be to start incorporating algae-derived carbon into products with a longer life cycle. For example, the building industry could become one of the greatest sinks for stored carbon by using algae-derived carbon products such as wall panels, foams and plastics [6].

Besides mitigating climate change, pivoting to biomanufacturing using algae-driven CCM offers additional industrial benefits. If we can select species/strains of algae that perform some of the chemical conversion steps, this reduces the industry chemical processes costs and limits the use of harsh polluting solvents. By transforming manufacturing into climate-positive biomanufacturing, using algae as cellular bio-factories has the potential to transform many industries and make them greener in the future to an extent that was unimaginable a few years ago [2,3,7].
